# Impact of Tai Chi exercise on multiple fracture-related risk factors in post-menopausal osteopenic women: a pilot pragmatic, randomized trial

**DOI:** 10.1186/1472-6882-12-7

**Published:** 2012-01-30

**Authors:** Peter M Wayne, Douglas P Kiel, Julie E Buring, Ellen M Connors, Paolo Bonato, Gloria Y Yeh, Calvin J Cohen, Chiara Mancinelli, Roger B Davis

**Affiliations:** 1 Division for Preventive Medicine, Brigham and Women's Hospital, Harvard Medical School, Boston, MA 02215, USA; 2 Institute for Aging Research, Hebrew SeniorLife, Harvard Medical School, 1200 Centre Street, Boston, MA 02131, USA; 3 Division of Gerontology, Beth Israel Deaconess Medical Center, Harvard Medical School, 330 Brookline Ave, Boston, MA, 02215, USA; 4 Department of Physical Medicine and Rehabilitation, Harvard Medical School, Spaulding Rehabilitation Hospital, Boston MA 02114, USA; 5 Division of General Medicine and Primary Care, Department of Medicine, Beth Israel Deaconess Medical Center, 330 Brookline Ave, Boston, MA, 02215, USA; 6 Harvard Vanguard Medical Associates, Internal Medicine, 133 Brookline Avenue, Boston, MA 02215, USA

## Abstract

**Background:**

Tai Chi (TC) is a mind-body exercise that shows potential as an effective and safe intervention for preventing fall-related fractures in the elderly. Few randomized trials have simultaneously evaluated TC's potential to reduce bone loss and improve fall-predictive balance parameters in osteopenic women.

**Methods:**

In a pragmatic randomized trial, 86 post-menopausal osteopenic women, aged 45-70, were recruited from community clinics. Women were assigned to either nine months of TC training plus usual care (UC) vs. UC alone. Primary outcomes were changes between baseline and nine months of bone mineral density (BMD) of the proximal femur and lumbar spine (dual-energy X-ray absorptiometry) and serum markers of bone resorption and formation. Secondary outcomes included quality of life. In a subsample (n = 16), quiet standing fall-predictive sway parameters and clinical balance tests were also assessed. Both intent-to-treat and per-protocol analyses were employed.

**Results:**

For BMD, no intent-to-treat analyses were statistically significant; however, per protocol analyses (i.e., only including TC participants who completed ≥ 75% training requirements) of femoral neck BMD changes were significantly different between TC and UC (+0.04 vs. -0.98%; P = 0.05). Changes in bone formation markers and physical domains of quality of life were also more favorable in per protocol TC vs. UC (P = 0.05). Changes in sway parameters were significantly improved by TC vs. UC (average sway velocity, P = 0.027; anterior-posterior sway range, P = 0.014). Clinical measures of balance and function showed non-significant trends in favor of TC.

**Conclusions:**

TC training offered through existing community-based programs is a safe, feasible, and promising intervention for reducing multiple fracture risks. Our results affirm the value of a more definitive, longer-term trial of TC for osteopenic women, adequately powered to detect clinically relevant effects of TC on attenuation of BMD loss and reduction of fall risk in this population.

**Trial Registration:**

ClinicalTrials.gov: NCT01039012

## Background

Fractures resulting from osteopenia (low bone mineral density (BMD)) are associated with significant long-term, morbidity and high medical costs [1,2]. Optimal interventions for osteopenic women are not yet well-defined [3]. Since life-long drug therapy is expensive with uncertain consequences and potential toxicities, non-pharmacologic therapy offers an attractive alternative. Current guidelines for osteopenia include the recommendation for regular exercise [4]. However, there is currently no consensus regarding the optimal types and regimens of exercise for treating low BMD, or for addressing other fracture-related risk factors relevant to women with osteopenia (e.g. poor balance, decreased muscle strength).

Tai Chi (TC) is a mind-body exercise that is growing in popularity in the U.S. and shows potential as an effective, safe and practical intervention for women with low BMD. A substantial body of research suggests TC training may reduce falls and associated risk factors [5-8]. A handful of studies have evaluated the direct effects of TC on BMD [9-13]. However, few studies are randomized trials, most have significant methodological limitations, and we are not aware of any trials that have evaluated the impact of TC on both BMD and postural control in osteopenic women [14,15].

We conducted a pilot randomized controlled trial to assess study feasibility and to preliminarily assess the effectiveness of TC combined with usual care, compared to usual care alone, for attenuating bone loss in post-menopausal osteopenic women. An embedded biomotion sub-study was also undertaken to evaluate if Tai Chi training could improve parameters of balance that have been associated with reduced fall risk. In this short trial with a modest sample size, BMD was not expected to show statistically significant changes, but was measured to detect treatment-related trends and to estimate an effect size for a future study. To provide interventions that simulate community-based TC programs and maximize external validity, we utilized a pragmatic design by allowing participants to choose from prescreened TC schools in the Greater Boston area.

## Methods

### Study Design

Details regarding study design are presented elsewhere [16]. A total of 86 post-menopausal osteopenic women were randomized in a 1:1 ratio to receive 9 months of TC training in addition to usual care, or to usual care alone (control group). Study participants randomized to usual care were offered a 3-month course of TC as a courtesy following the trial. Primary outcomes assessed at 9 months were dual-energy X-ray absorptiometry (DXA) measures of BMD of the femoral neck, total hip, and lumbar spine, and serum levels of C-terminal telopeptide of type I collagen (CTX) and osteocalcin (OSC), biomarkers of bone resorption and formation, respectively. Outcomes assessed in the biomotion sub-study included two sway parameters measured during quiet standing (sway velocity and stabilogram ellipse area), and two clinical tests (tandem walk balance test and repeated chair rise). Institutional Review Boards of Harvard Medical School, Beth Israel Deaconess Medical Center, Hebrew SeniorLife, and Partner's HealthCare approved this study. All recruitment and intervention protocols took place between September 2008 and January 2010.

### Study population

Participants were recruited from a large network of Boston area clinics serving approximately 300,000 members. Inclusion criteria were: 1) Women ages 45-70 y; 2) BMD T-scores of the femoral neck or trochanter and/or spine between -1.0 and -2.5; 3) post-menopausal without menses for ≥ 12 months; 4) exercise no more than 5 days a week on average for more than 60 minutes per day. Exclusion criteria were: 1) Osteoporosis (T-score < -2.5) at any site; 2) prior or current use of medication that influence bone metabolism (e.g. steroids, anticoagulants); 3) prior or current use of medications that modify bone metabolism (e.g. bisphosphonates, selective estrogen receptor modulators); 4) use of calcium supplements above 1200 mg); 5) current or prior year use of estrogen or calcitonin; 6) malignancies other than skin cancer; 7) diagnosis of anorexia along with a BMI of < 17.5; 8) conditions that cause secondary osteoporosis (e.g. Cushing's syndrome, hyperparathyroidism); 9) tobacco use in past year; 10) physical or mental disabilities precluding active study participation; 11) scheduling limitations that would preclude participation; 12) practice of TC within past 2 years.

We used mailings to invite women aged 45-70 who had eligible DXA scan results during the prior two years. Those interested who passed a phone screen were scheduled for a visit at the Beth Israel Deaconess Medical Center Clinical Research Center where they provided written informed consent to participate in the study, and a DXA scan was conducted to confirm eligibility. Randomization assignments were given to participants following baseline testing; these were generated by computer using a permuted block design with a variable block size.

### Interventions

Participants randomized to both groups were encouraged to follow standard of care for osteopenic women (including daily calcium, vitamin D, and regular exercise) as prescribed by their primary physicians. Participants in the TC group received nine months of TC training in addition to usual care. All TC interventions were administered at one of seven pre-screened schools within the Greater Boston area that met specific guidelines described elsewhere [16]. Instructors were asked to teach using the same approach and protocols employed for non-study, community participants. Study participants were asked to attend a minimum of two classes per week for the first month, and one class per week for eight months thereafter (minimum class duration of one hour). They were asked to practice an additional two times per week during the first month, and three times per week thereafter (minimum of 30 minutes per session), which could be home practice or additional classes at their school. Thus, participants were asked to participate in TC training a total of 99.5 hours over the 9 month interventions.

### Outcome measures

All primary outcome measures were assessed by study staff blind to treatment assignment. BMD of the hip and spine was measured by DXA using a QDR 4500 Discovery densitometer (Hologic, Inc., Waltham, MA) in the array (fan beam) mode by the same technician at each visit. At the screening visit, subjects underwent a single measurement of the left hip and spine. Nine-month follow-up measurements were analyzed using the "comparison" feature of the standard Hologic APEX 2.3 analysis program for matched identical regions of interest, and all serial scans were reviewed for quality and scan analysis by one of the investigators.

Bone resorption was assessed using serum CTX [17,18]. Serum OSC was used as a marker for bone formation [17,19]. We hypothesized that TC would have antiresorptive effects and expected to see decreases in markers of both resorption and formation, since the two are often tightly coupled [20]. All specimens were obtained in the morning and following a 12 hour fast to minimize diurnal variability.

The Medical Outcomes Survey Form (SF-36) health status survey was used to assess overall health-related quality of life [21]. We also evaluated menopausal-specific symptoms using the Menopause Quality of Life instrument (MENQOL) [22,23]. Physical activity was assessed using the Seven-Day Physical Activity Recall (PAR) [24,25], recently modified to include strength and flexibility activities [24,26]. The PAR estimates daily total energy expenditure (kcal/kg/d).

Attendance at TC classes was recorded and home practice was tracked using a weekly practice log. Adverse events were monitored through systematic monthly safety calls conducted by study staff.

Expectation regarding the beneficial impacts of TC for bone health was assessed for all participants at baseline [27]. For participants in the TC group, satisfaction with the intervention was assessed by asking the following four statements (5 point scale; 1 strongly agree, 5 strongly disagree) at 3 and 9 month visits: 'Overall, I am satisfied with my TC experience in the study'; 'Overall, I am satisfied with my TC school'; 'Overall, I am satisfied with the TC teachers I am training with'; 'I would recommend the TC program I am enrolled in to a friend or relative.'

### Biomotion sub-study methods and outcomes

A total of 16 participants, 8 from each group, volunteered to participate in an embedded sub-study to evaluate the impact of TC on balance-related outcomes. All balance outcomes were assessed in the Motion Analysis Laboratory (MAL) of Spaulding Rehabilitation Hospital. All tests were performed barefoot and completed in one session of approximately 3 hours. Biomotion instrumentation in the MAL includes an eight-camera motion analysis system (VICON, Oxford, UK) and force platforms (AMTI, Watertown, MA). The force platforms were used to collect ground reaction forces, from which we estimated center of pressure (CoP) data and parameters of balance control during quiet standing.

Tests of quiet standing were conducted while subjects stood on a force platform for 40 s with arms by their side, feet shoulder-width apart and their eyes closed. Tests with eyes closed are known to be more "provocative" than tests performed with eyes open. Each subject completed 10 trials; subjects were allowed to take a short break and sit down after 5 trials. Raw trajectories were extracted from the force plates and smoothed with a 4^th ^order Butterworth filter with cut-off frequency of 25 Hz. A scatter plot of the anteroposterior (AP) and mediolateral (ML) displacement of the CoP, called a stabilogram, was analyzed for each trial. Balance control during the quiet standing trials was characterized via traditional sway parameters including average sway velocity (mm/sec), total stabilogram ellipse area (mm^2^), and anterior-posterior (AP) sway range. These parameters have shown to be predictive of falls [28,29].

Subjects also performed two widely used clinical tests; tandem walking [30] and repeated chair rise [31,32]. These tests have been shown to discriminate balance ability, be predictive of falls, and to be related to muscle strength [31,33-35]. During the tandem walking test, subjects were instructed to walk so that at each step the toes of the back foot touched the heel of the front foot. Subjects were asked to walk 10 meters as quickly as possible. During the chair rise test, subjects were asked to sit on the same standard chair so that their back was in contact with the back rest. They were then instructed to stand up and sit down again 10 times without stopping at a comfortable speed. Both clinical tests were repeated three times and the average of all trials was used. Subjects were given rest breaks between each test and trials of a test.

### Statistical Analysis, Sample Size and Power

Primary clinical outcomes were percentage change from baseline to 9 months in DXA, CTX and OSC. All primary analyses were conducted according to the intention-to-treat paradigm. For primary and secondary outcome measures, we used Wilcoxon rank sum tests to compare change from baseline between treatment groups. Secondary pre-specified analyses included a per protocol evaluation. Participants were considered per protocol if they adhered to a minimum of 75% of required classes (43 hours total) and home practice (56.5 hours total), which was defined as 74.6 hours of total training over 9 months.

Based on results of Yamazaki's study of walking for women with osteopenia [36] and allowing for 15% loss to follow-up, we estimated that 43 women per group would provide 60% power to detect a between-group difference in the change in bone mineral density (DXA) of 0.9% and 80% power to detect differences in change in CTX of 20%.

## Results

### Recruitment feasibility, protocol adherence, and safety

**Figure 1:**
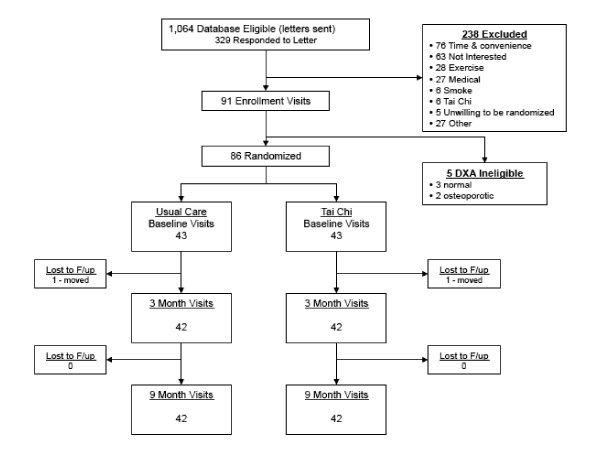
**Study flow diagram**.

Figure 1 summarizes participant study flow. Forty-two of 43 (98%) individuals in each group completed baseline and 9-month follow-up protocols. Adherence with TC interventions was variable. Twenty-six patients (60%) were considered per protocol. Average combined total training time (i.e., class plus home) over the 9-month intervention was 83.2 hours (median = 93.2 h; range = 0 to 226 h) for all participants randomized to TC and 120.6 hours (median = 107.3; range 82 to 226 h) for those who were per protocol. Interventions were administered at six of the seven pre-screened schools that were provided as options to participants, with 59% of those attending classes based on the Wu style of TC and 41% attending classes based on the Yang style of TC. Participant self-reported satisfaction with their TC intervention was very high; median satisfaction scores for all questions were 1.0 (highest score) at 3 and 9 months.

### Baseline characteristics

Randomization resulted in comparable TC and control groups at baseline (Table 1). For all variables, values for the subset of per-protocol participants were comparable to those in the larger sample, minimizing some sources of bias in post-hoc comparisons between Usual Care and TC compliant groups.

**Table 1:** Characteristics of study participants at baseline randomized to both the usual care control group and the Tai Chi intervention.

Variable	Randomized to Usual Care (n = 43)	Randomized to Tai Chi (n = 43)	Per Protocol Tai Chi (n = 26)
Age in years (mean ± SD (range))	60.4 ± 5.3 (46-70)	58.8 ± 5.6 (43-70)	59.1 ± 4.9 (51-68)
Race: n (%)			
White	36 (84)	37 (86)	21 (88)
African American	2 (5)	4 (9)	2 (8)
Asian	3 (7)	1 (2)	0 (0)
Other	2 (5)	1 (2)	1 (4)
Education: n(%)			
High School/GED	4 (9)	1 (2)	0
Some College	5 (12)	3 (7)	1 (4)
College	13 (30)	15 (35)	8 (33)
Graduate degree	21 (49)	24 (56)	15 (63)
BMI in kg/m^2 ^(mean ± SD)	24.5 ± 4.0	25.8 ± 4.2	25.8 ± 3.8
Years post-menopause, n (%)			
1-7	18 (42)	24 (56)	12 (50)
> 7	18 (42)	12 (28)	7 (29)
unknown	7 (16)	7 (16)	5 (21)
Calcium supplementation, n (%)			
Yes	34 (79)	34 (79)	19 (79)
No	8 (19)	7 (16)	3 (13)
unknown	1 (2)	2 (5)	2 (8)
BMD in g/cm^2 ^(mean ± SD):			
Femoral neck	0.692 ± 0.068	0.680 ± 0.063	0.685 ± 0.074
Total hip	0.837 ± 0.077	0.829 ± 0.073	0.832 ± 0.079
Spine	0.901 ± 0.128	0.898 ± 0.070	0.898 ± 0.076
Serum bone turnover markers (mean ± SD)			
CTX ng/ml	0.603 ± 0.231	0.554 ± 0.259	0.594 ± 0.300
OSC ng/ml	15.52 ± 4.94	16.29 ± 6.01	17.11 ± 7.02
Physical activity (PAR, kcal/kg/d) (mean ± SD)	144.5 ± 43.6	128.0 ± 38.4	123.9 ± 26.9
SF-36 (mean ± SD):			
Physical	53.2 + 5.6	53.5 + 6.1	51.9 + 6.8
Mental	55.8 + 4.8	52.0 + 7.7	53.7 + 7.0
Expectancy (mean ± SD)*:			
Q.1: 'Improving your health'	3.79 + 0.80	3.88 + 1.12	3.92 + 1.38
Q.2: 'Recommend to a friend'	3.67 + 1.13	3.60 + 1.69	3.67 + 1.74
Q.3: 'Makes sense to you'	4.09 + 0.97	4.19 + 1.10	4.21 + 1.32

* Expectancy values based on 5 point Likert scale; higher values indicate greater expectancy that Tai Chi will positively impact health

Also shown are data for the subset of participants randomized to Tai Chi whose compliance placed them in the "per protocol" group (i.e. ≥ 75% compliant with class and home practice). All continuous variables are means ± standard deviation. Categorical variable are described with frequency count followed by percentages..

### Intervention related changes in outcomes

**Figure 2:**
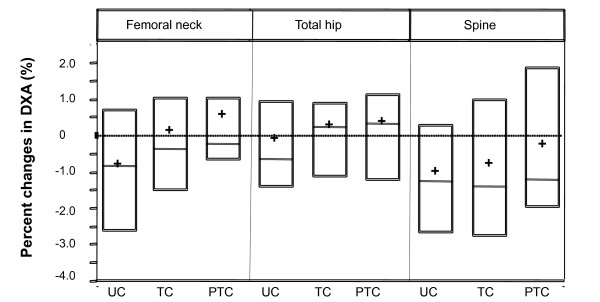
**Treatment related changes (%) in bone mineral density of the femoral neck, total hip, and lumbar spine assessed using dual energy x-ray absorptiometry (DXA)**. Boxes represent 25^th ^and 75^th ^percentiles, horizontal lines represent median values, and + sign represent mean values. Data are presented separately for participants randomized to Usual Care (UC) and Tai Chi (TC), as well as the subset of those in the Tai Chi group that were Per-Protocol (PTC).

TC tended to attenuate bone loss at all sites measured (Figure 2, Table 2). At the femoral neck, women randomized to Usual Care lost an average of approximately 1% (-0.98%) over the 9-month period. In contrast, those randomized to TC experienced no loss (-0.01%), and for the TC per protocol subset, there was a slight increase in BMD (+0.04%). The intent-to-treat analysis comparing randomized groups was not statistically significant (P = 0.23); however, the secondary comparison between the per protocol and Usual Care groups did differ significantly (P = 0.05).

**Table 2:** Changes from baseline to 9 months in primary outcomes, bone mineral density (BMD) and bone turnover markers.

Variable	Randomized to Usual Care (n = 43)		Randomized to Tai Chi (n = 43)		Per Protocol Tai Chi (n = 26)	
	Baseline	9 Months	Baseline	9 Months	Baseline	9 Months
**BMD**						
Femoral Neck (mean ± SD) (g/cm^2^) % change (median [Q1, Q3])	.692 ± .068	.685 ± .069 -0.85 [-2.58, 0.70]	.680 ± .063	.681 ± .063 -0.39 [-1.48, 1.02] *p = 0.24	.685 ± .074	.688 ± .075 -0.23 [-0.64, 1.02] *p = 0.05
Total Hip (mean ± SD) (g/cm^2^) % change (median [Q1, Q3])	.837 ± .077	.835 ± .078 -0.64 [-1.39, 0.92]	.829 ± .073	.832 ± .074 0.23 [-1.12, 0.89] *p = 0.26	.832 ± .079	.834 ± .079 0.35 [-1.20, 1.13] *p = 0.32
Spine (mean ± SD) (g/cm^2^) % change (median [Q1, Q3])	.901 ± .128	.891 ± .132 -1.27 [-2.67, 0.28]	.898 ± .070	.889 ± .069 -1.38 [-2.72, 0.99] *p = 0.98	.898 ± .076	.894 ±.072 -1.21 [-1.95, 1.88] *p = 0.38
						
**Bone Turnover Markers**						
CTX (mean ± SD) (ng/ml) % change (median [Q1, Q3])	.603 ± .231	.629 ± .310 2.05 [-15.33, 23.19]	.554 ± .259	.569 ± .248 0.68 [-17.94, 24.19] *p = 0.99	.594 ± .30	.552 ± .266 -7.17 [-25.77, 12.56] *p = 0.21
OSC (mean ± SD) (ng/ml) % change (median [Q1, Q3])	15.52 ± 4.94	16.50 ± 4.82 4.30 [0.00, 11.80]	16.29 ± 6.01	16.38 ± 5.72 1.94 [-10.03, 12.87] *p = 0.35	17.11 ± 7.02	16.23 ± 5.96 -3.55 [-13.61, 7.23] *p = 0.03

* p-value for comparison with Usual Care based on the Wilcoxon rank sum test.

Baseline and 9 month values represent mean values ± standard deviations. Change scores are summarized with median values and 25^th ^(Q1) and 75^th ^(Q3) percentiles. P-values indicate comparisons between Usual Care ground and Tai Chi and Per Protocol groups, based on the Wilcoxon rank sum test.

Average magnitudes of 9-month BMD changes in the total hip and total spine ranged from more than a 1% loss (e.g., Usual Care total spine) to slight increases in (e.g., per protocol total hip). At both sites, trends paralleled the femoral neck with the per protocol TC group resulting in the greatest attenuation of BMD loss; however, comparisons between groups were not statistically different. The observed effect sizes for femoral neck, spine and hip were 0.34, 0.22, and 0.08, respectively.

### Bone turnover markers

**Figure 3:**
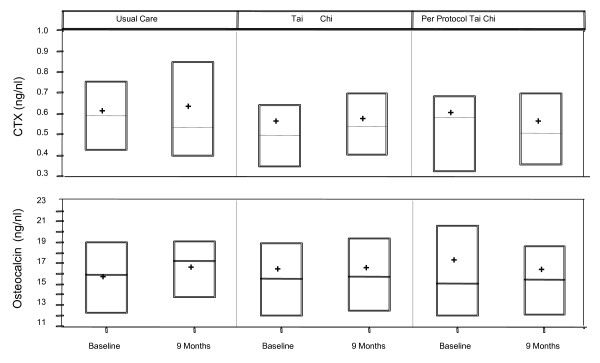
**Treatment related changes in serum markers of bone resorption (C-terminal cross linking telopeptide of type I collagen (CTX)) and bone formation (osteocalcin)**. Boxes represent 25^th ^and 75^th ^percentiles, horizontal lines represent median, and + sign represent mean values.

Treatment related changes for markers of both resorption (CTX) and formation (OSC) were modest and trended in the same direction (Figure 3, Table 2). In the Usual Care group, average serum concentrations of both CTX and OSC increased (4.3% and 6.3%, respectively). In contrast, in the per protocol TC group, both CTX and OSC decreased (-7.1% and -5.1%, respectively). Values in the randomized TC group were intermediate. No intent-to-treat comparisons were statistically significant, but the difference in the magnitude of OSC changes between the Usual Care and per protocol TC group were statistically significant (P = 0.03).

### Quality of life and physical activity

Subscales related to physical function for both the SF-36 and MENQOL showed trends towards modest improvement in women who practiced TC per protocol (Table 3). Post-hoc analyses comparing this subgroup to the Usual Care group were significant at 3 months for the SF-36 physical scale (P = 0.02) and at 9 months for MENQOL physical subscale (P = 0.03). Similar, but statistically non-significant trends were observed for all other MENQOL subscales. Self-reported physical activity (PAR) increased over the course of the trial in all groups (Table 3).

**Table 3:** Physical and mental health related quality of life (SF 36), overall menopause related quality of life (MENQOL) and subscales, and physical activity recall (PAR).

Variable	Randomized to Usual Care (n = 43)			Randomized to Tai Chi (n = 43)			Per Protocol Tai Chi (N = 26)		
	Baseline	3 Months	9 Months	Baseline	3 Months	9 Months	Baseline	3 Months	9 Months
SF 36 Physical	53.2 ± 5.6	53.3 ± 6.0	53.1 ± 6.4	53.5 ± 6.1	52.9 ± 6.8	52.6 ± 7.7	51.9 ± 6.8	54.7 ± 5.3*	53.7 ± 6.6
								.	
SF 36 Mental	55.8 ± 4.8	54.6 ± 5.6	54.0 ± 8.4	52.0 ± 7.7	52.1 ± 10.1	52.6 ± 8.5	53.7 ± 7.0	53.6 ± 8.1	54.0 ± 6.6
									
MENQOL subscales									
Vasomotor	1.78 ± 1.08	1.85 ± 1.46	1.79 ± 1.34	2.02 ± 1.36	1.89 ± 1.31	1.86 ± 1.24	1.83 ± 1.20	2.08 ± 1.53	1.76 ± 1.14
									
Psychosocial	1.83 ± 0.93	1.91 ± 1.04	2.01 ± 1.51	2.27 ± 1.20	2.16 ± 1.22	2.33 ± 1.17	2.13 ± 1.27	1.91 ± 1.01	1.98 ± 1.01
									
Physical	1.82 ± 0.68	1.91 ± 0.89	2.02 ± 0.97	1.97 ± 0.81	1.94 ± 0.68	1.96 ± 0.73	2.00 ± 0.96	1.77 ± 0.53	1.71 ± 0.58**
									
Sexual	1.73 ± 1.37	1.75 ± 1.58	1.77 ± 1.34	1.96 ± 1.34	1.84 ± 1.27	1.81 ± 1.41	1.68 ± 1.25	1.51 ± 0.99	1.50 ± 1.24
									
MENQOL Total	1.79 ± 0.74	1.84 ± 0.95	1.90 ± 0.93	2.07 ± 0.87	1.97 ± 0.78	1.99 ± 0.88	1.94 ± 0.92	1.84 ± 0.76	1.74 ± 0.76
									
PAR (kcal/kg/d)	144.5 ± 43.6	166.5 ± 55.7	160.1 ± 51.9	128.0 ± 38.4	151.4 ± 46.0	153.0 ± 38.3	123.9 ± 26.9	155.5 ± 47.6	156.4 ± 42.7
									

* Indicates change from base line to 3 months significantly different from Usual Care group at P = 0.02 (Wilcoxon rank sum test)

** Indicates change from base line to 9 months significantly different from Usual Care group at P = 0.03 (Wilcoxon rank sum test)

Values represent group means ± standard deviation at baseline, 3 and 9 months.

### Biomotion sub-study results

Fifteen of the 16 sub-study participants completed follow-up assessments. Baseline characteristics of this sub-group, including median age, BMI, time since menopause, and BMD were comparable to those for the total randomized study population. Nine months of TC training resulted in improved balance compared to Usual Care (Table 4). Median change values for all sway parameters improved for the TC group but worsened for Usual Care; group differences were statistically significant for average sway velocity (P = 0.027) and AP sway range (P = 0.014) and stabilogram ellipse area differences approached significance (P = 0.060). Clinical tests of balance (tandem walk) and function (repeated chair rise) showed relatively greater improvement in TC vs. Usual Care, but these trends were not statistically significant.

**Table 4:** Changes from baseline to 9 months in balance related outcomes based on a subgroup of patients randomized to usual care and Tai Chi.

Variable	Usual Care (n = 7)		Tai Chi (n = 8)		P-Values*
	Baseline	9 Months	Baseline	9 Months	
**Biomotion sway parameters**					
Avg. sway velocity (mean ± SD) (mm/sec) % change (median [Q1, Q3])	10.75 ± 3.29	12.55 ± 5.26 0.92 (0.01, 2.49)	9.30 + 1.83	9.01 ± 1.83 -0.24 (-0.66, 0.51)	P = 0.027
AP sway range (mean ± SD) (mm) % change (median [Q1, Q3])	22.53 ± 7.56	25.14 ± 9.86 2.27 (-1.55, 4.95)	24.45 ± 4.92	21.49 ± 5.41 -3.19 (-4.58, -1.88)	P = 0.014
Stabilogram ellipse area (mean ± SD) (mm2) % change (median [Q1, Q3])	184.6 ± 180.2	234.5 ± 254.5 31.73 (8.08, 41.27)	155.4 ± 62.4	134.1 ± 71.2 -37.72 (-59.59, -29.40)	P = 0.060
**Clinical Balance and Function Test**					
Tandem walk (sec) % change (median [Q1, Q3])	34.43 ± 9.14	31.37 ± 12.48 -1.82 (-9.86, 2.42)	42.75 ± 8.51	33.25 ± 7.82 -8.83 (-13.8, -5.46)	P = 0.116
Repeated chair rise (sec) % change (median [Q1, Q3])	25.95 ± 6.55	23.91 ± 2.37 2.40 (-7.26, 2.93)	26.47 ± 4.39	23.73 ± 2.77 -2.39 (-4.97, -0.87)	P = 0.232

Baseline and 9 month values represent mean values ± standard deviations. Change scores are summarized with median values and 25^th ^(Q1) and 75^th ^(Q3) percentiles. P-values indicate comparisons between Usual Care ground and Tai Chi, based on the Wilcoxon rank sum test.

### Adverse Events

No serious adverse events were reported in the trial. A total of nine minor adverse events were reported, seven in the TC group and two in the control group. Reports in both groups were largely musculoskeletal related (e.g. shoulder or back pain); none in the TC group were attributed directly to TC training.

## Discussion

Few randomized trial have evaluated the potential of Tai Chi to impact multiple fracture-related risk factors in osteopenic women. In this short trial with a modest sample size, BMD and balance-related outcomes were not expected to show statistically significant changes in response to TC, but was measured to detect treatment-related trends and to estimate an effect size for a future study. We observed a clinically relevant trend of TC training attenuating bone loss. Trends towards improved BMD, a reduction in bone turnover, and better health related quality of life in the TC vs. Usual Care were not statistically significant for any variable when evaluated with an intent-to-treat analysis. However, secondary analyses comparing per protocol TC participants to Usual Care for BMD of the femoral neck, physical domains related to quality of life, and osteocalcin levels indicated statistically significant positive effects. Additionally, results from our biomotion sub-study suggest clinically and statistically relevant improvements in balance parameters previously shown to be associated with fall risk. Our results affirm the value of a future, more definitive trial of TC for osteopenic women, provide the preliminary required data for determining sample size for appropriate statistical power for such a trial, and contribute to a growing literature evaluating TC for bone health and fall-related fracture risk.

Prior research on the effects of TC on BMD in post-menopausal osteopenic women is limited [14,15]. Cross-sectional studies including elderly women suggest long-term TC practitioners have higher BMD than age-matched sedentary controls [37,38], and have slower rates of post-menopausal BMD decline [11]. One RCT in post-menopausal women observed that DXA measures of BMD at the lumbar spine significantly increased (1.81%) following 10 months of TC while sedentary controls decreased (1.83%) [39]. A second RCT observed that for older women, 12 months of TC training resulted in maintenance of total hip BMD levels when compared to a non-exercise control that lost 2.25% of total hip BMD [40].

This study uniquely extends our understanding of the potential impact of TC on women's bone health. Prior studies have included women with BMDs ranging from normal to severely osteoporotic. Because the effects of antiresorptive treatment on bone turnover and changes in BMD may vary with severity of bone loss [41], our study specifically informs the value of TC to post-menopausal women with a firm diagnosis of osteopenia. Second, we report on an ethnically diverse Western population. Patterns of postmenopausal BMD loss vary with respect to race and ethnicity, and nearly all prior RCTs evaluating TC for BMD have been conducted in Asia. Our results are in concordance with a recently completed U.S. study of osteopenic women that reported 6 months of Tai Chi improved multiple markers of bone health including higher levels of bone-specific alkaline phosphotase (BAP), higher ratios of BAP to tartrate-resistant acid phosphotase, and elevated levels of serum parathyroid levels [42,43]. Third, in contrast to previous RCTs that targeted relatively sedentary populations, we included women who were relatively active since exercise is a standard recommendation for osteopenia. Our results suggest that benefits of TC on the skeleton are not limited to sedentary individuals.

The Erlangen Fitness Osteoporosis Prevention Study (EFOPS) [44] was a RCT comparing a graded multipurpose exercise program to a usual care control group. After 1 year, magnitudes of differences between the exercise vs. control group in the femoral neck (-0.8% vs. -1.8%) and total hip (-0.3% vs. -0.8%) were comparable to the trends we observed after 9 months of TC training; however, improvement in BMD of the total spine were more dramatic in EFOPS (+1.3% vs. -1.2%). After two years, exercise attenuated any further loss at all sites, whereas cumulative bone loss in the non-exercise control was approximately 2.9%, 1.7%, and 2.3% at the femoral neck, total hip and total spine, respectively [45]. It is plausible that extending the period of TC training from 9 months to two years would result in continued attenuation of bone loss, as observed in the EFOPS trial. Based on both clinical relevance, feasibility, and our preliminary analyses, we believe a future longer-term TC study is warranted, and estimate that a moderate size trial of approximately 200 participants would be adequately powered to detect the differences in BMD observed in the EFOPS trial.

Independent of changes in BMD, TC may be of benefit to women with low bone density because of its positive effect on fall risk and postural control. Numerous randomized trials suggest TC training can directly reduce prevalence of falls [5,8]. Other studies suggest TC positively impacts factors associated with falls including multiple sway parameters [46-48], clinical balance tests [49], musculoskeletal strength and flexibility [50-52], and fear of falling [48,53,54]. One study including only osteopenic women reported positive effects of 6 months of Tai Chi on one gait stride length, but not on dynamic posturography or clinical measures of balance [55]. However, this and the majority of other studies have only included older adults with more limited postural control. The results from our biomotion substudy suggest that the balance-related benefits of Tai Chi observed in older populations may also extend to relatively younger and healthier osteopenic women. Confirmation of our results in a larger and longer-term trial would suggest that, in combination with its modest effects of BMD, TC is a potentially valuable intervention for prevention of falls and fall-related fractures in post-menopausal osteopenic women, and goes beyond most fracture interventions that target only the skeleton.

Our intervention was not based on a single fixed training protocol, but rather relied on the diversity of protocols provided naturalistically in pre-screened, long-standing community TC schools [56]. As such, our use of a pragmatic intervention affords high external validity, applying not just to one specific TC training protocol, but rather to the diversity of protocols encountered in typical community-based programs. However, our use of naturalistic interventions reduces the internal validity of our study. This intervention heterogeneity may necessitate larger samples to increase statistical power [57].

Our choice of a usual care control followed the overarching practical goal of our study--to evaluate the potential benefits to osteopenic women of adding TC to usual care. However, this choice limits the conclusion we can draw, particularly regarding the mechanisms underlying the trends we observed in BMD, postural control and QOL. Because we did not control for group psychosocial interactions, time, and expectancy of receiving a therapy, it is possible that we measured only placebo effects. By not comparing TC to other active interventions that might offer comparable doses of weight bearing, or resistance and flexibility training, we cannot ascribe which aspect(s) of TC contributed to its therapeutic effects.

TC is a complex, multi-component intervention, and it is possible that it impacts bone remodeling via multiple processes. Motion analysis studies of TC practitioners have reported that compared to normal gait, lower extremity movements during TC have: longer cycle duration and single-leg stance time; greater ankle, knee, and hip joint motion; larger lateral body shift; distinct plantar pressure distributions; and greater and unique patterns of lower extremity muscle activation [58-62]. Compared to normal gait, TC has also been shown to have larger peak shear forces in the ankle, knee and hip joints, and larger peak moments in the knee and hip joints [63]. Cross-sectional studies of elders have shown that numerous aspects of lower extremity muscle strength and endurance are comparable to joggers [52], and other randomized studies have reported that TC training can favorably reorganize lower extremity neuromuscular patterns, resulting in reduced excessive hip compensation and more efficient gait [64].

### Study Limitations

A few other limitations of our study are important to acknowledge. First, subjects were not blinded to their intervention group. We attempted to minimize the potential effects of disappointment in the usual care group by offering free TC classes at the end of study. Second, our relatively small sample size does not discount the possibility that the results we observed were due to chance. Third, the 9-month duration of our study is relatively short from both the perspective of providing an adequate dose of TC, as well as the sensitivity of DXA to detect BMD changes. However, this study was conceived as a pilot study. As such, it has provided the data to inform a future more definitive study that will employ a larger sample and longer period of intervention and observation. Finally, while participant retention and compliance with outcomes protocols was high (loss to follow-up was 2%), adherence to TC training was lower than expected. Earlier TC studies have reported higher protocol adherence rates [65-69]. The lower rates we observed in this study may be due to our relatively long, 9-month intervention. Only a handful of TC studies to date have evaluated interventions longer than six months, and most have been less than four months. Lower adherence may also result from our use of a pragmatic design. Prior studies with higher adherence typically utilized study-trained instructors, fixed cohorts of participants sharing a common medical condition, and were based in medical settings with participants having regular contact with study stuff. Greater and more structured contact with study participants and Tai Chi school staff will be required to improve adherence in a future trial.

## Conclusion

We observed a clinically relevant trend of TC attenuating bone loss and improving quality of life in postmenopausal osteopenic women. These trends were statistically significant in "per protocol" secondary analyses. A change in BMD of 1-2% is clinically significant as the risk for fracture doubles for each SD lower BMD and a gain of 2-4% in BMD from pharmacologic therapy results in close to a 50% reduction in fracture risk [70]. We also observed statistically significant improvements in fall-predictive measures of postural control. In combination with previous studies suggesting TC may attenuate bone loss in elderly women, and research in older adults suggesting that TC may reduce fall-related fracture risk by improving postural control and preventing falls [6,7], our results affirm the value of a future, more definitive trial of TC for fracture prevention in osteopenic women.

## Competing interests

Peter Wayne is a sole proprietor of a community based Tai Chi school in the Boston area, which served as one of the sites for this study, but received no financial compensation. Douglas Kiel has received grants from Amgen, Merck, Novartis, Wyeth, and Hologic; served as a consultant for Amgen, Merck, Novartis, GSK, Philips Lifeline, Wyeth, P&G, and Lilly; and has been a speaker for Novartis, Merck, and GSK (although not as of July 2010). All other authors declare that they have no competing interests.

## Authors' contributions

PW conceived of the study and has oversight of its conduct, and along with, DK, RD, PB, EC and JB contributed to finalization of study design. DK had oversight for all markers of BMD. PB and CM were responsible for collection and analysis of biomotion outcomes. EC served a study coordinator. CC was responsible for patient identification. RD was responsible for statistical analyses. All authors read and approved the final manuscript.

## Pre-publication history

The pre-publication history for this paper can be accessed here:

http://www.biomedcentral.com/1472-6882/12/7/prepub
